# Supplementary material for: The extracellular matrix protects Bacillus subtilis colonies from Pseudomonas invasion and modulates plant co-colonization
Source: Nat Commun. 2019 Apr 23;10:1919. doi: 10.1038/s41467-019-09944-x (PMC6478825; doi:10.1038/s41467-019-09944-x)
Supplement: Supplementary file 1 — Supplementary Information [file 41467_2019_9944_MOESM1_ESM.pdf]

The extracellular matrix protects *Bacillus subtilis* colonies from *Pseudomonas* invasion and modulates plant co-colonization

Molina-Santiago et al.,

Supplementary material

Supplementary Figures

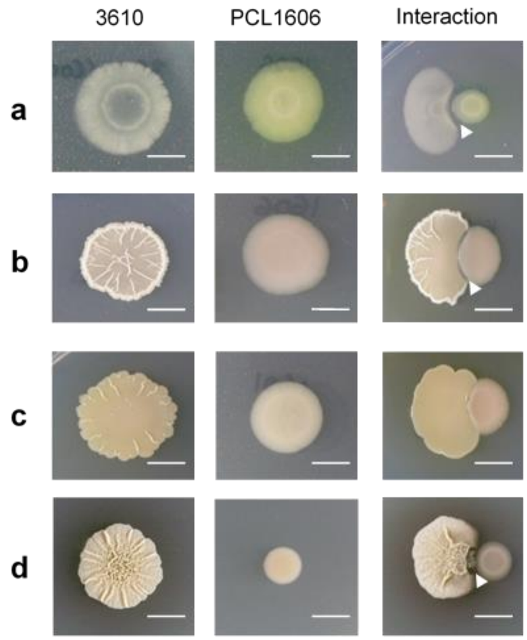

Suppl. Fig 1. Single colony growth and pairwise interactions between PCL1606 and 3610 in different culture mediums after 72 h of growth. (a) Growth in King's B medium; (b) growth in LB medium; (c) growth in TY medium; and (d) growth in Msgg medium. White arrows indicate the inhibition area of PCL1606 against 3610. Scale bar = 5mm.

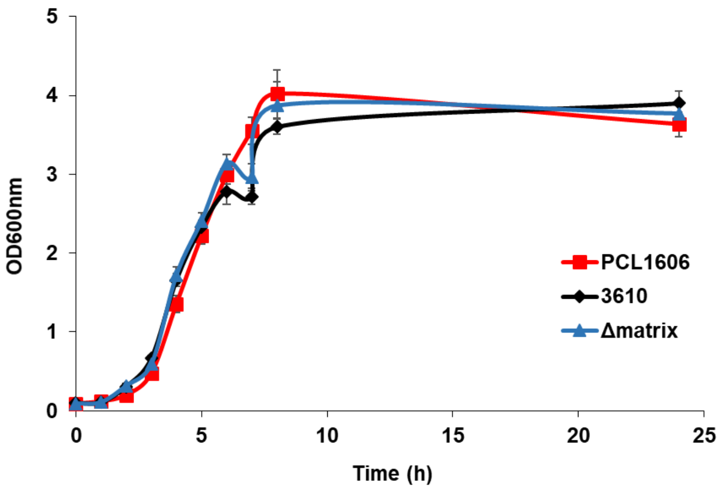

Suppl. Fig. 2. Growth curves of PCL1606 (red line), 3610 (black line) and Δmatrix (blue line) growing in LB medium. Average values of three biological replicates are shown, with error bars representing SD. Source data are provided as a Source Data file.

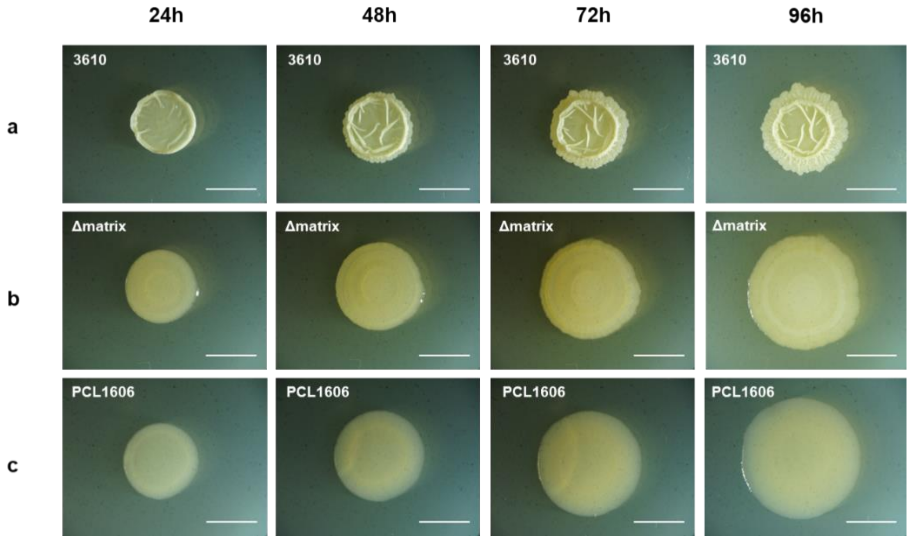

Suppl. Fig. 3. (a, b and c) Time-courses of *B. subtilis* (a) 3610, (b) Δmatrix and (c) *P. chlororaphis* PCL1606 colony morphologies at 24 h, 48 h, 72 h and 96 h when growing alone in LB medium. Scale bar = 5 mm.

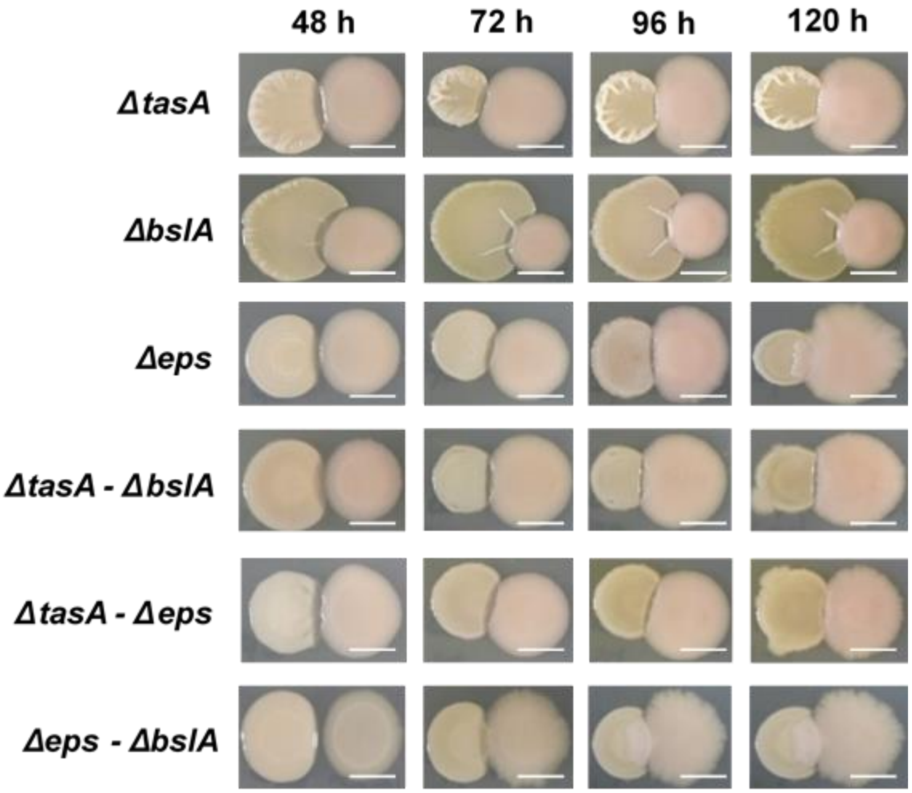

Suppl. Fig. 4. Time-courses of the interactions between PCL1606 (right side colonies) and *Bacillus* strains (left side colonies): ΔTasA, ΔBslA, ΔEps, ΔTasA-ΔBslA, ΔTasA-ΔEps, and ΔEps-ΔBslA at 48 h, 72 h, 96 h and 120 h when growing as single-cultures in LB medium. Scale bar = 5 mm.

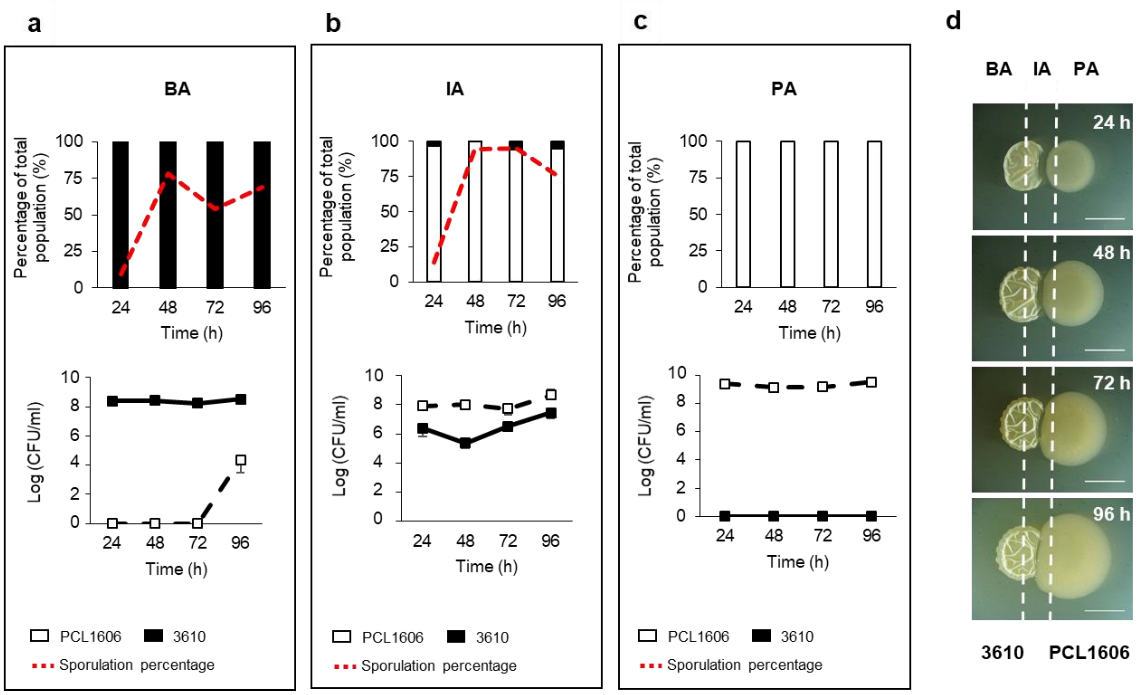

Suppl. Fig 5. a, b and c panels show (upper part) percentages and (bottom part) Log (CFU/ml) of PCL1606 (empty bars and squares) and 3610 (black bars and squares) in three different sections (BA [3610 area], IA [Intermediate area] and PA [PCL1606 area]) of the interaction at different time-points (24 h, 48 h, 72 h, 96 h). Sporulation rates are shown as red lines. (d) Scheme of the interactions and the sections: BA (3610 area), IA (Intermediate area) and PA (PCL1606 area). Scale bar = 5 mm. Cells from the three sections of the interaction were collected, sonicated and completely resuspended, diluted and plated to determine colony forming units (CFU), sporulation rates and percentages of each species over the total bacterial population in the interaction. Average values of three biological replicates are shown, with error bars representing SD. Source data are provided as a Source Data file.

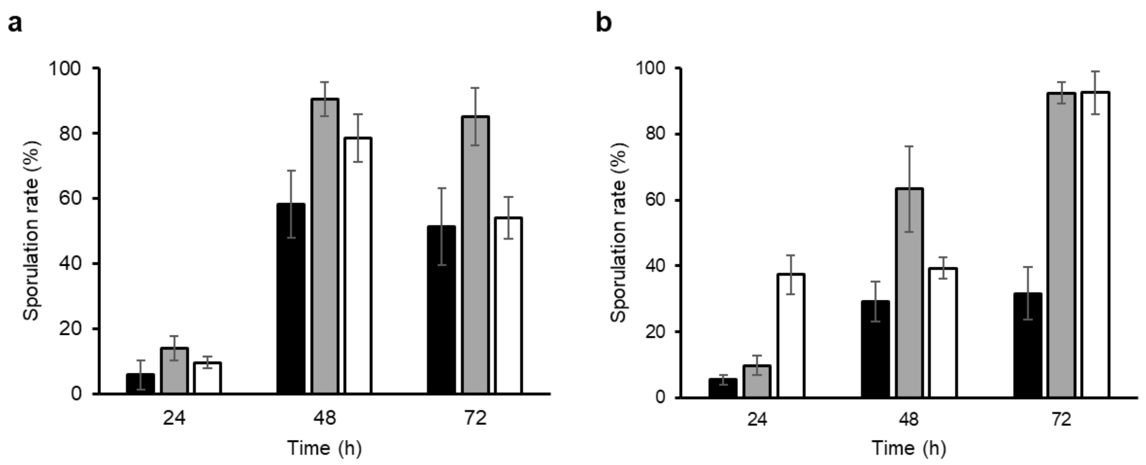

Suppl. Fig 6. Sporulation percentages of (a) 3610 and (b) Δmatrix growing in LB medium during 24 h, 48 h and 72 h of growth. Black bars indicate sporulation percentage of single colonies, grey bars indicate sporulation percentages of *B. subtilis* strains in the intermediate area (IA) after interaction with PCL1606, and white bars indicate sporulation percentages of *B subtilis* strains in the Bacillus area (BA) after interaction with PCL1606 (n =3). Average values of three biological replicates are shown, with error bars representing SD. Source data are provided as a Source Data file.

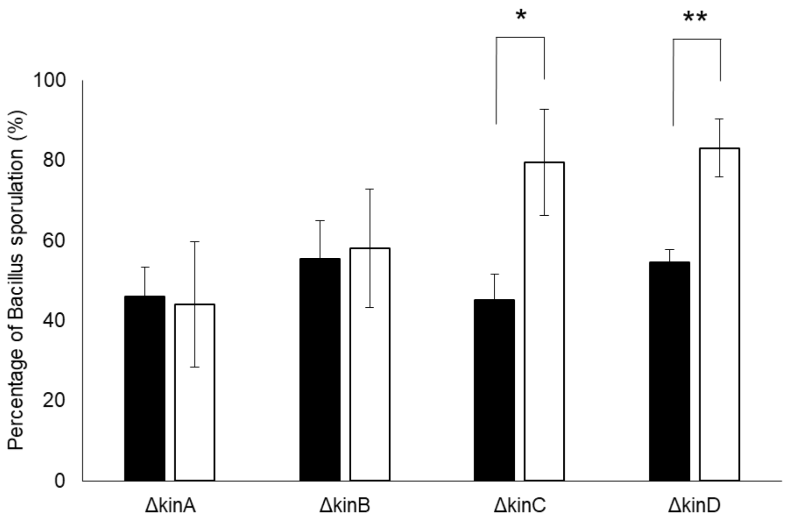

Suppl. Fig. 7. Percentage of sporulation in *B. subtilis* 3610 strains mutated in *kinA*, *kinB*, *kinC* and *kinD* histidine kinases when growing alone (black bars) and in the interaction with PCL1606 (empty bars) at 72 h of growth in LB medium. Average values of three biological replicates are shown, with error bars representing SD. **P*-value < 0.05, ***P*-value < 0.01 (*t*-student). Source data are provided as a Source Data file.

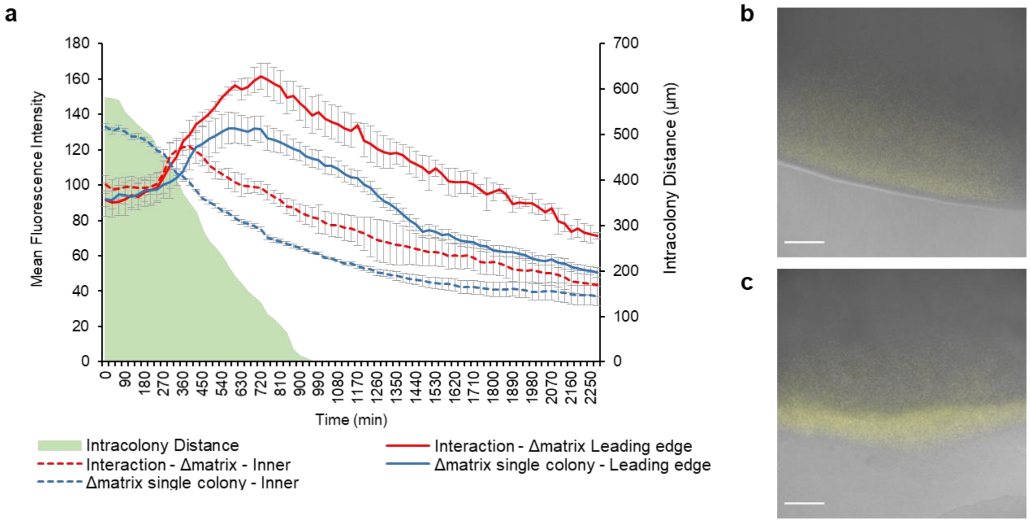

Suppl. Fig. 8. (a) Comparison of the mean of fluorescence intensity of the *sppB* promoter of a Δmatrix single colony (blue lines) and of Δmatrix in the interaction with PCL1606 (red lines). Dashed lines indicate sporulation in the inner of the colony while continuous lines indicate sporulation at the leading edge of the colony. Green area indicates the intracolony distance between Δmatrix and PCL1606 during time. Average values of three biological replicates are shown, with error bars representing SD. (b) and (c) show the sporulation of *sppB* promoter in (b) Δmatrix single colony and (c) Δmatrix – PCL1606 interaction. Scale bar = 100 µm. Source data are provided as a Source Data file.

24h

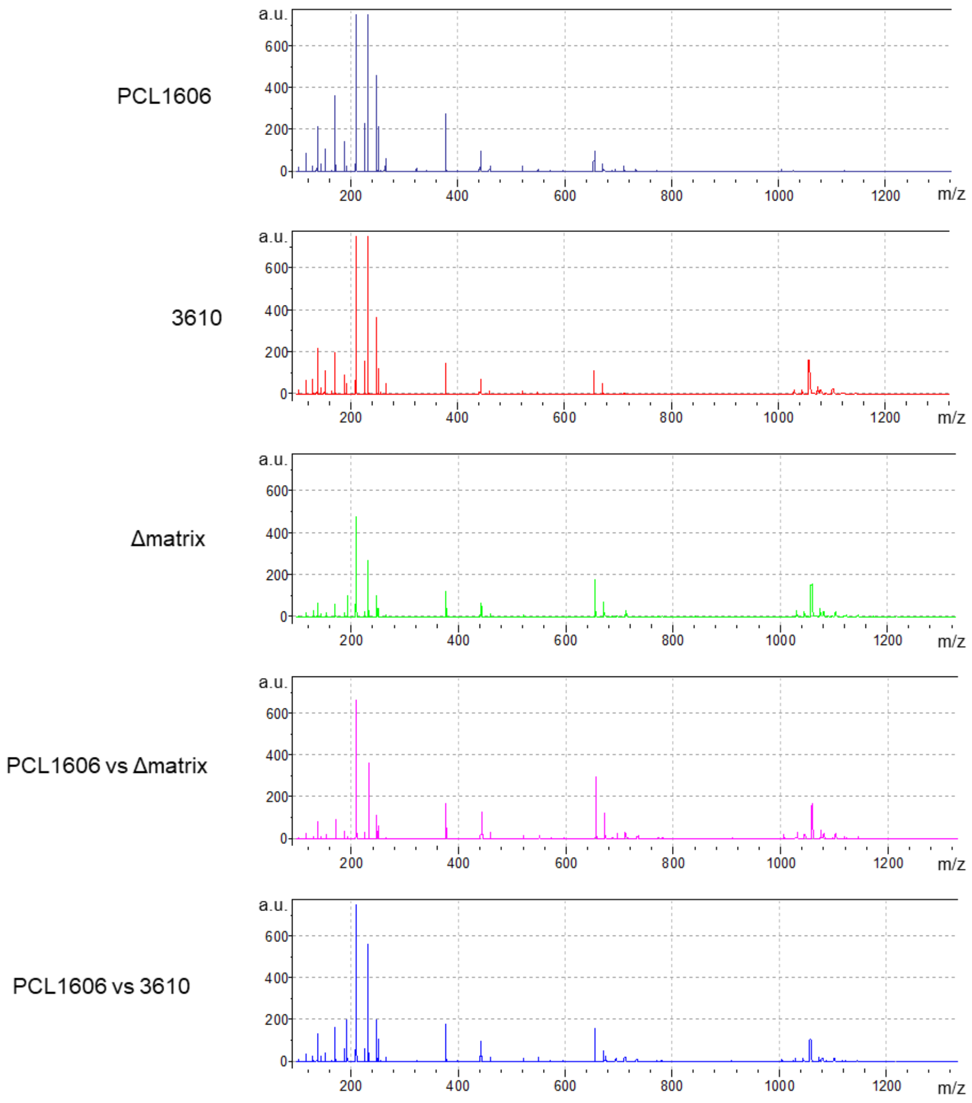

Suppl. Fig. 9. Average mass spectrum obtained by MALDI-TOF MSI of PCL1606, 3610 and Δmatrix growing alone and in interactions after 24 h of culture in LB medium.

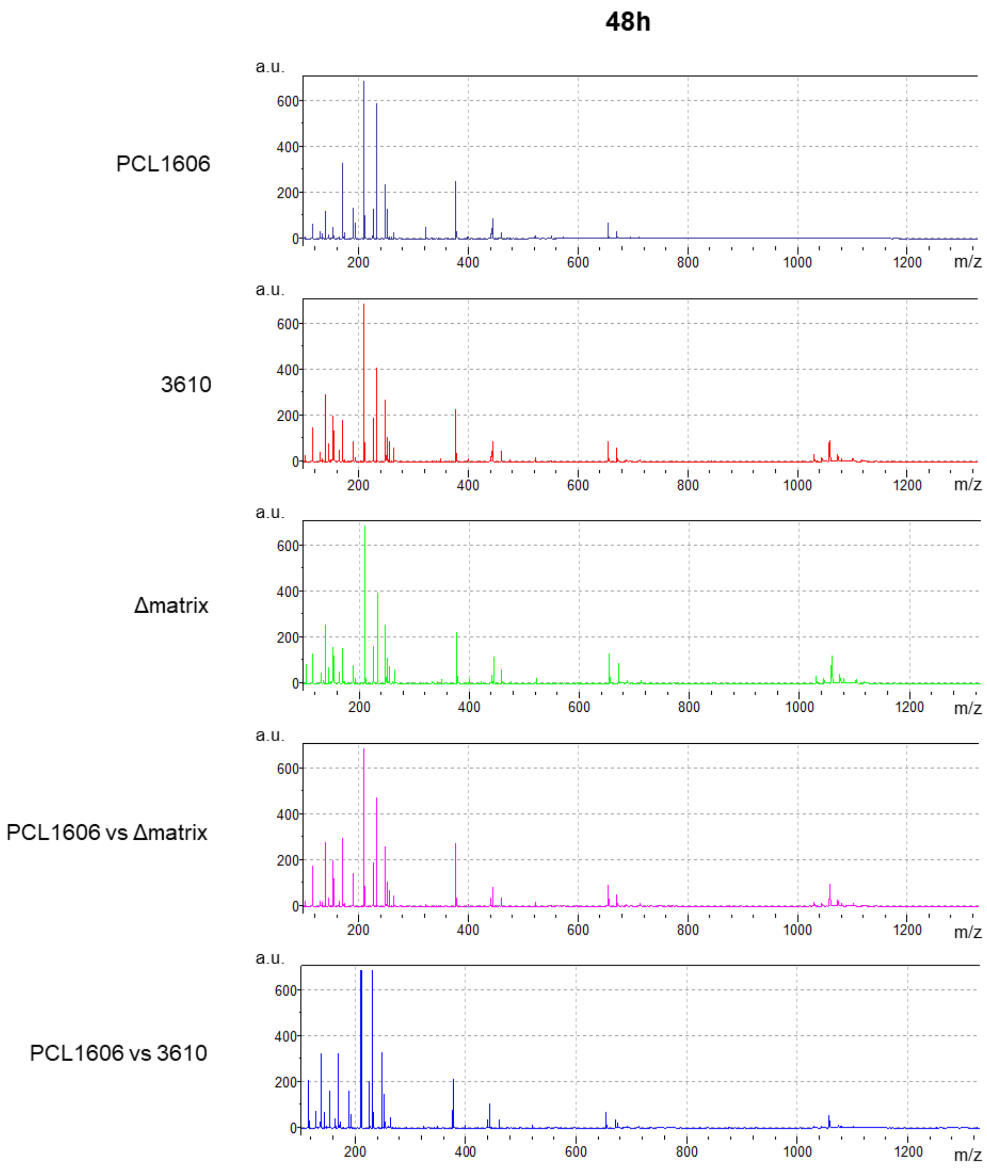

Suppl. Fig. 10. Average mass spectrum obtained by MALDI-TOF MSI of PCL1606, 3610 and Δmatrix growing alone and in interactions after 48 h of culture in LB medium.

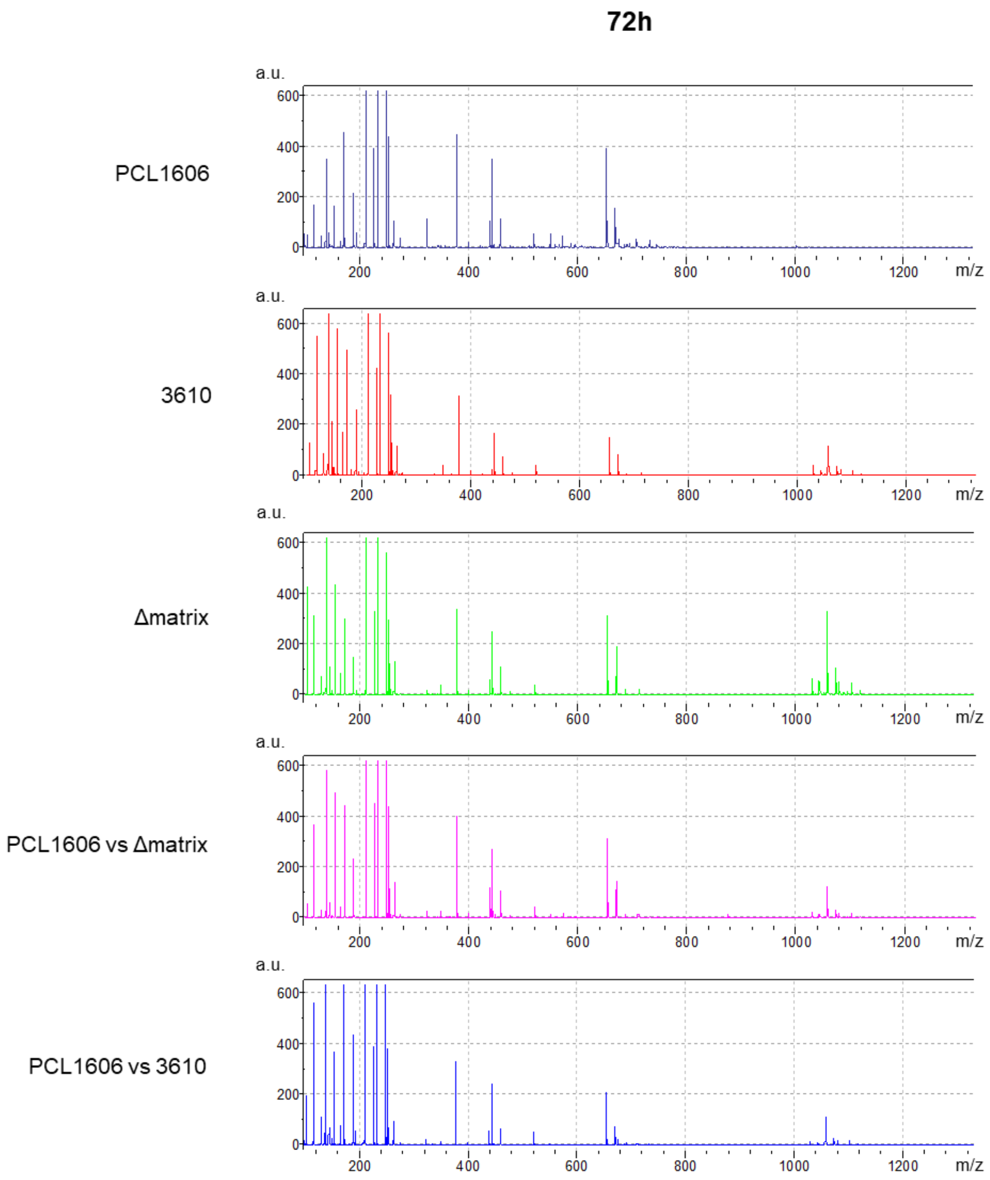

Suppl. Fig. 11. Average mass spectrum obtained by MALDI-TOF MSI of PCL1606, 3610 and Δmatrix growing alone and in interactions after 72 h of culture in LB medium.

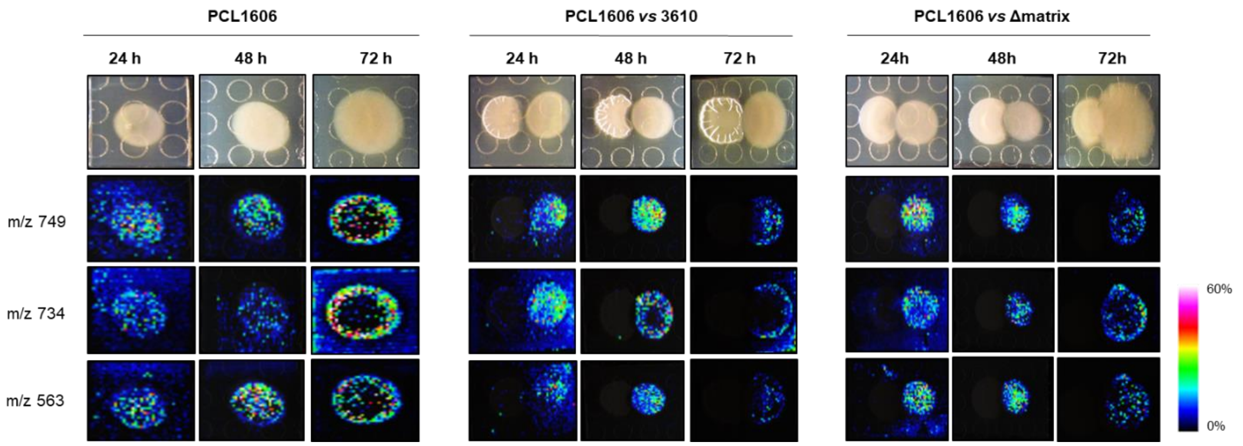

Suppl. Fig. 12. MALDI-TOF MSI heatmaps of selected molecules during time-lapse experiments (24 h, 48 h, 72 h) of PCL1606 growing alone and in interaction with *Bacillus* strains. As a representation of the similar metabolite distribution between samples, molecules with m/z of 749, 734 and 563 are shown.

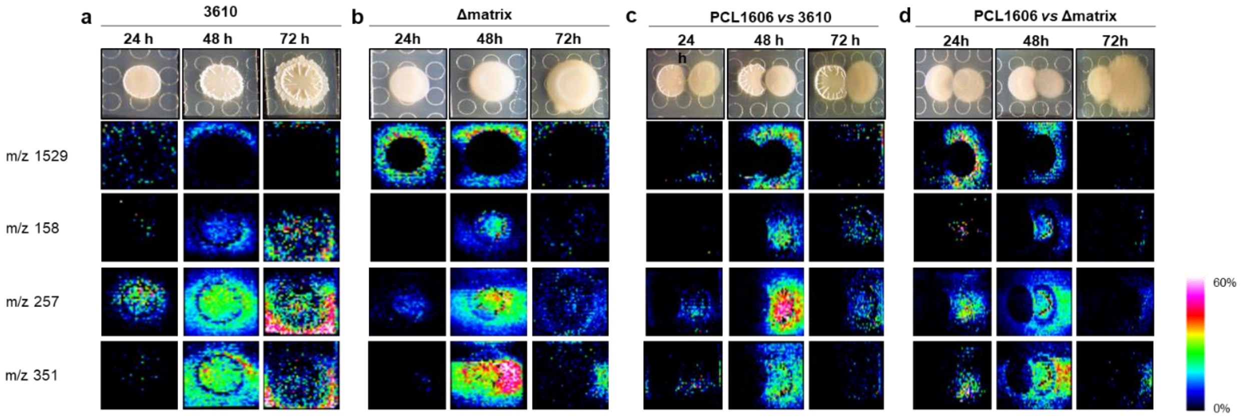

Suppl. Fig. 13. MALDI-TOF MSI heatmaps of selected molecules during time-lapse experiments (24 h, 48 h, 72 h) of (a) 3610 and (b) Δmatrix growing alone and in (c) and (d) interaction with PCL1606. Molecules with similar distribution patterns are shown: m/z = 1529, 158, 257 and 351.

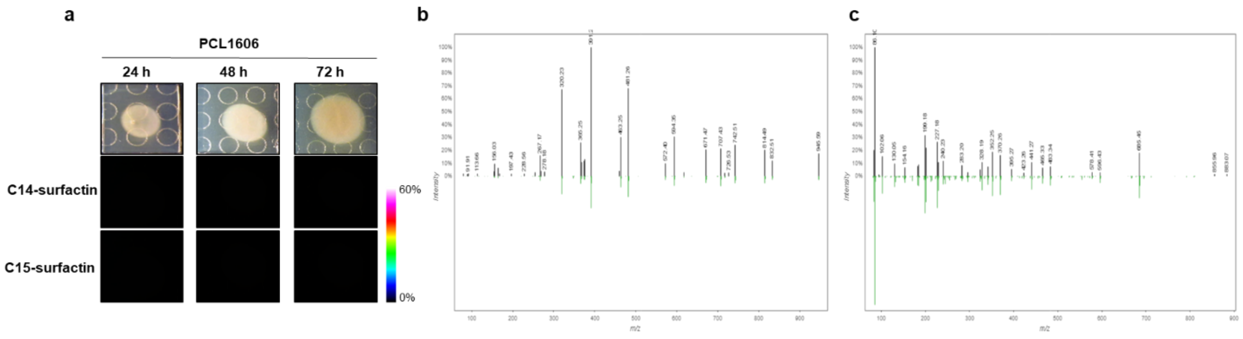

Suppl. Fig. 14. (a) MALDI-TOF MSI time-lapse experiments (24 h, 48 h, 72 h) showing the non-production of C14 and C15-surfactin isoforms by PCL1606. (b) and (c) Mirror plots of the fragmented spectra for (b) C14-surfactin and (c) C15-surfactin isoforms. Green fragmented spectra correspond to the spectra found in GNPS database.

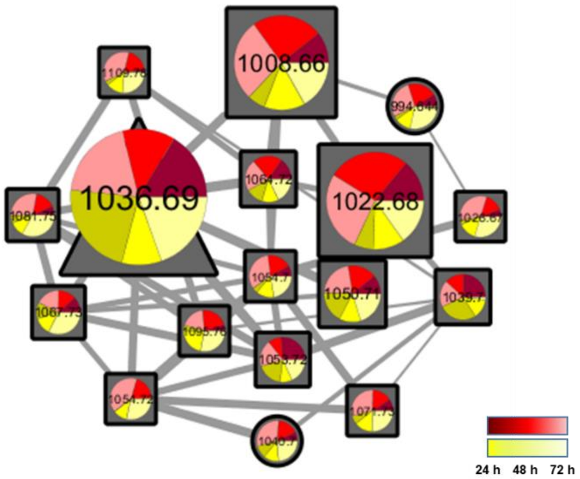

Suppl. Fig. 15. View of the molecular networking results of the surfactins cluster ($M+H^+$). Red portions indicate surfactins in 3610 – PCL1606 interactions while yellow portions indicate surfactins in the interaction Δmatrix – PCL1606 at 24 h, 48 h and 72 h of interaction (from dark to light colors, respectively). Size is directly related with abundance of the molecules. C13, C14 and C15-surfactin isoforms correspond to m/z 1008, 1022 and 1036 ($M+H^+$), respectively. Triangles represent compounds that are found at GNPS database, while squares indicate analogs and circles indicate not analog compounds.

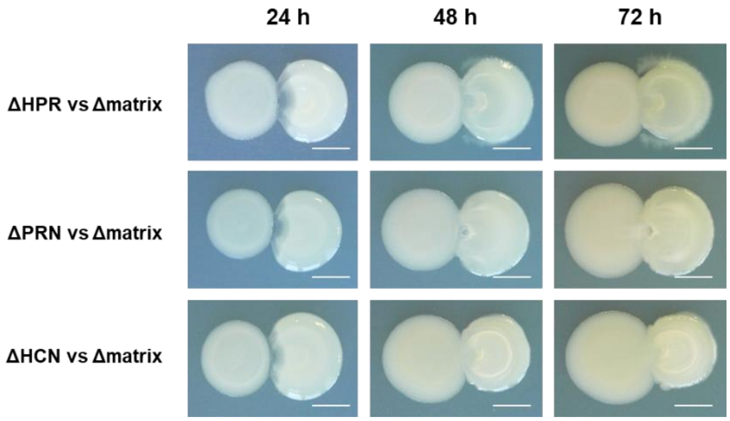

Suppl. Fig. 16. Time-course interaction experiments between PCL1606 mutants in secondary metabolites and Δmatrix. Mutants of PCL1606 were unable to produce (a) HPR (2-hexyl-5-propyl-resorcinol), (b) PRN (pyrrolnitrin), and (c) HCN (hydrogen cyanide). Right colonies in the interaction are Δmatrix while left colonies are PCL1606 mutants. Scale bar = 5 mm.

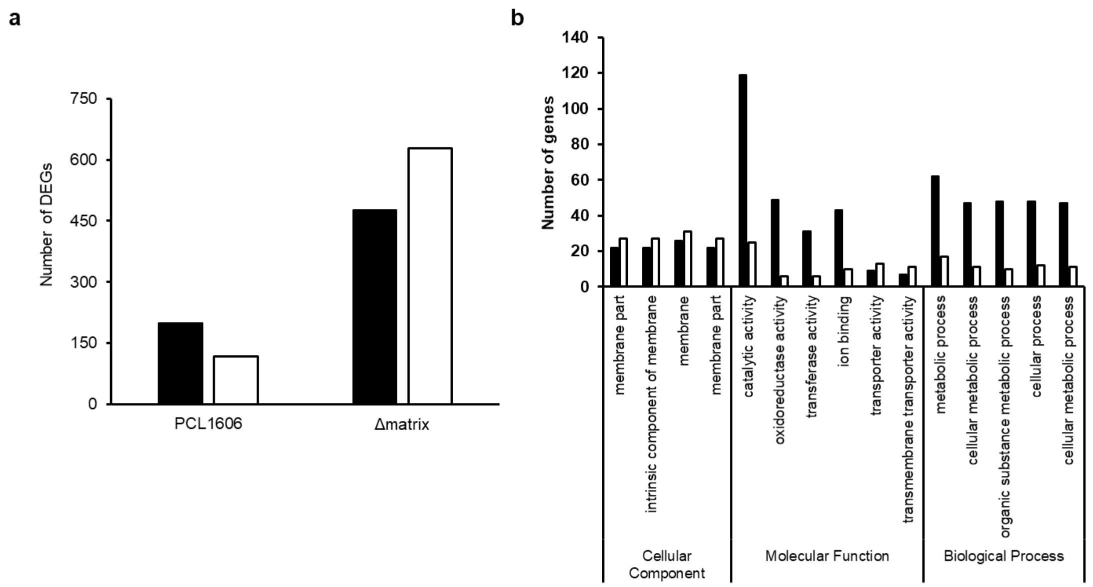

Suppl. Fig. 17. Gene ontology (GO) terms analysis of the differentially expressed genes during the interaction between PCL1606 and Δmatrix. (a) Number of genes differentially expressed (DEGs) after 72 h of interaction of PCL1606 and Δmatrix. Black bars indicate induced genes and empty bars indicate repressed genes. (b) GO terms differentially expressed in PCL1606. Black bars indicate induced GO terms while empty bars indicate induced GO terms.

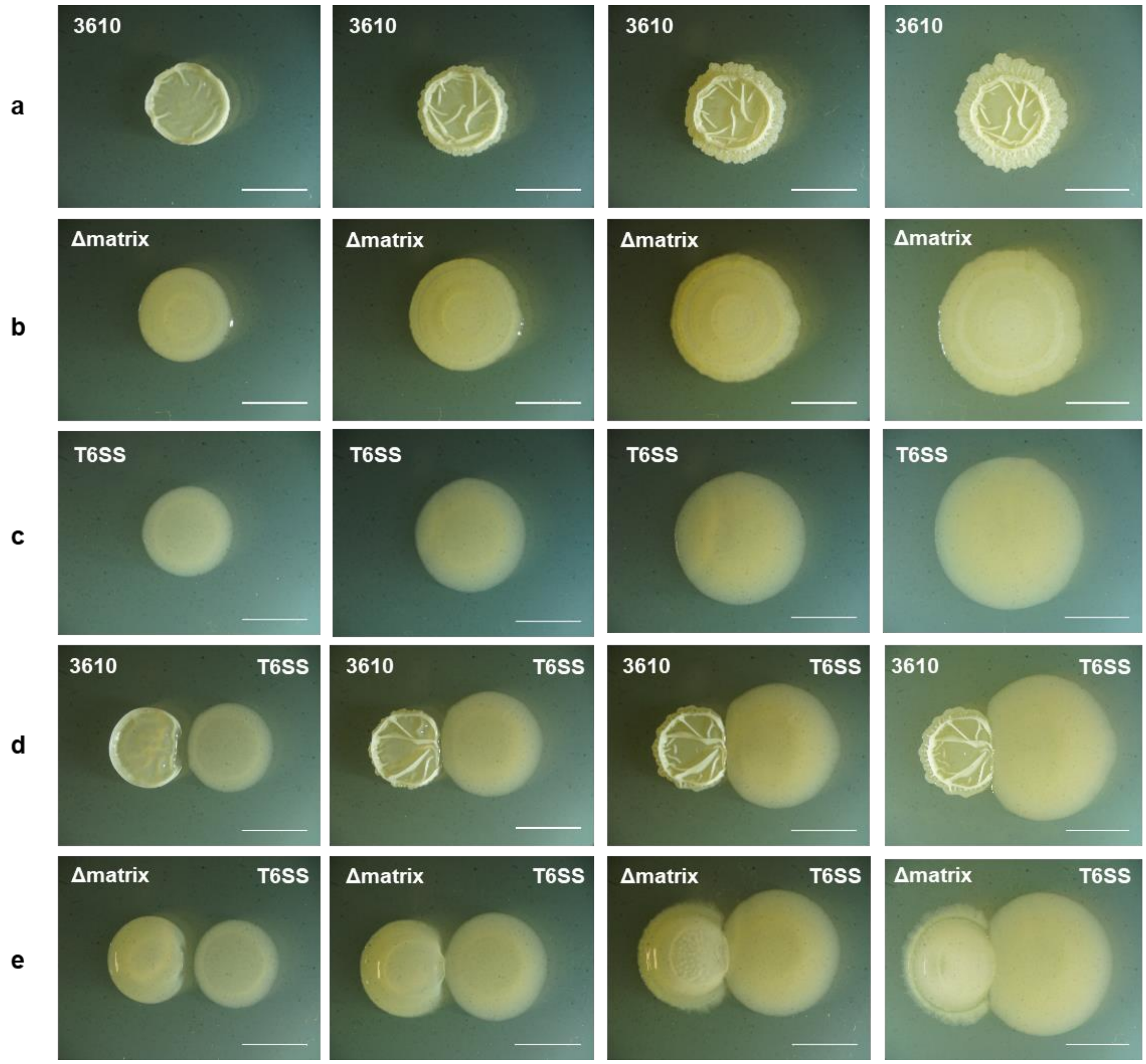

Suppl. Fig. 18. (a, b and c) Time-courses of *B. subtilis* (a) 3610, (b) Δmatrix and (c) ΔT6SS colony morphologies at 24 h, 48 h, 72 h and 96 h when growing alone in LB medium. (d and e) Time-courses of the interactions (d) 3610-ΔT6SS and (e) Δmatrix-ΔT6SS at 24 h, 48 h, 72 h and 96 h growing in LB medium. Left colonies are *Bacillus* strains and right colonies are ΔT6SS. Scale bar = 5 mm.

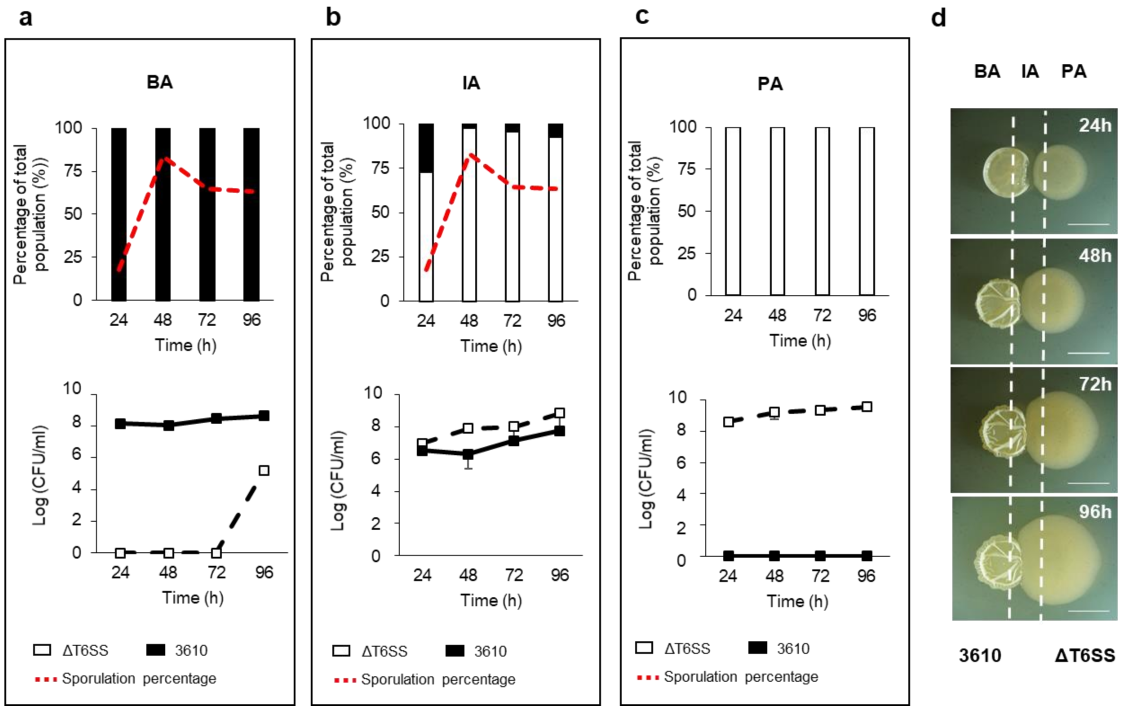

Suppl. Fig. 19. a, b and c panels show (upper part) percentages and (bottom part) Log (CFU/ml) of ΔT6SS (empty bars and squares) and 3610 (black bars and squares) in three different sections (BA [3610 area], IA [Intermediate area] and PA [ΔT6SS area]) of the interaction at different time-points (24 h, 48 h, 72 h, 96 h). Sporulation rates are shown as red lines. (d) Scheme of the interactions and the sections: BA (3610 area), IA (Intermediate area) and PA (ΔT6SS area). Scale bar = 5 mm. Cells from the three sections of the interaction were collected, sonicated and completely resuspended, diluted and plated to determine colony forming units (CFU), sporulation rates and percentages of each species over the total bacterial population in the interaction. Average values of three biological replicates are shown, with error bars representing SD. Source data are provided as a Source Data file.

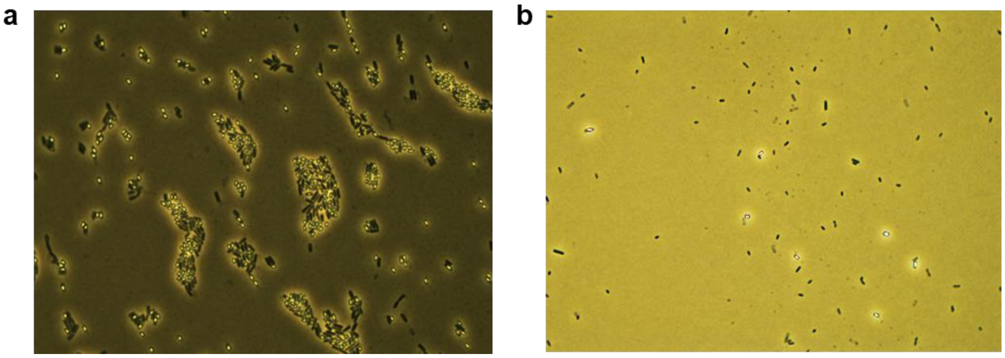

Suppl. Fig. 20. Microscopic images of the interaction area between PCL1606 and Δmatrix at (a) 48 h and (b) 72 h of interaction. Spores can be observed as white brilliant spots. (a) shows the spores in the initial moments of the first contact between colonies while in (b) spores can be observed with an increase number of PCL1606 bacterial cells.

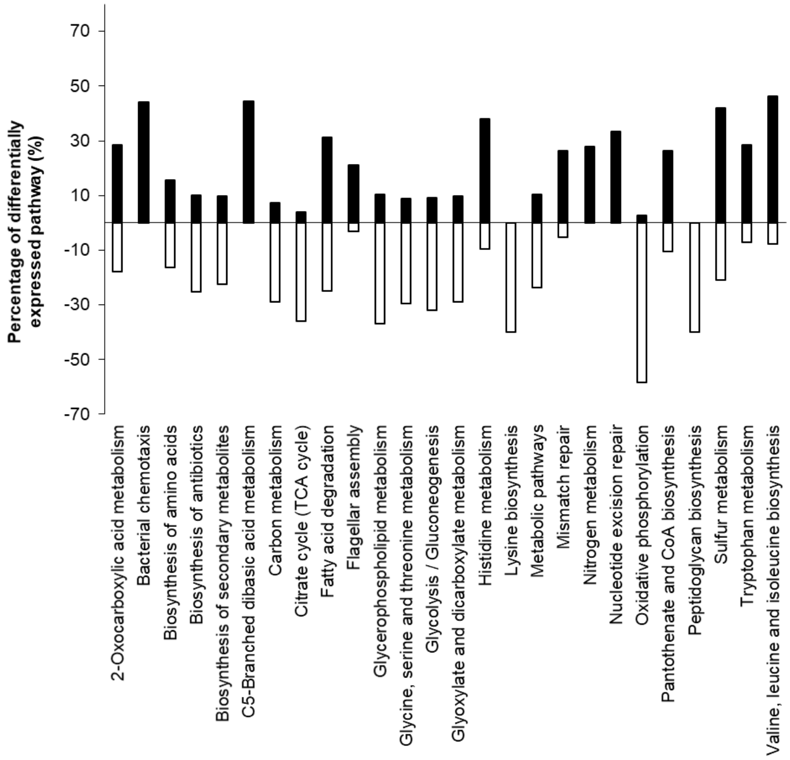

Suppl. Fig. 21. KEGG pathway analysis of the Δmatrix genes induced (black bars) and repressed (empty bars) after 72 h of interaction with PCL1606 (*P*-value < 0.05).

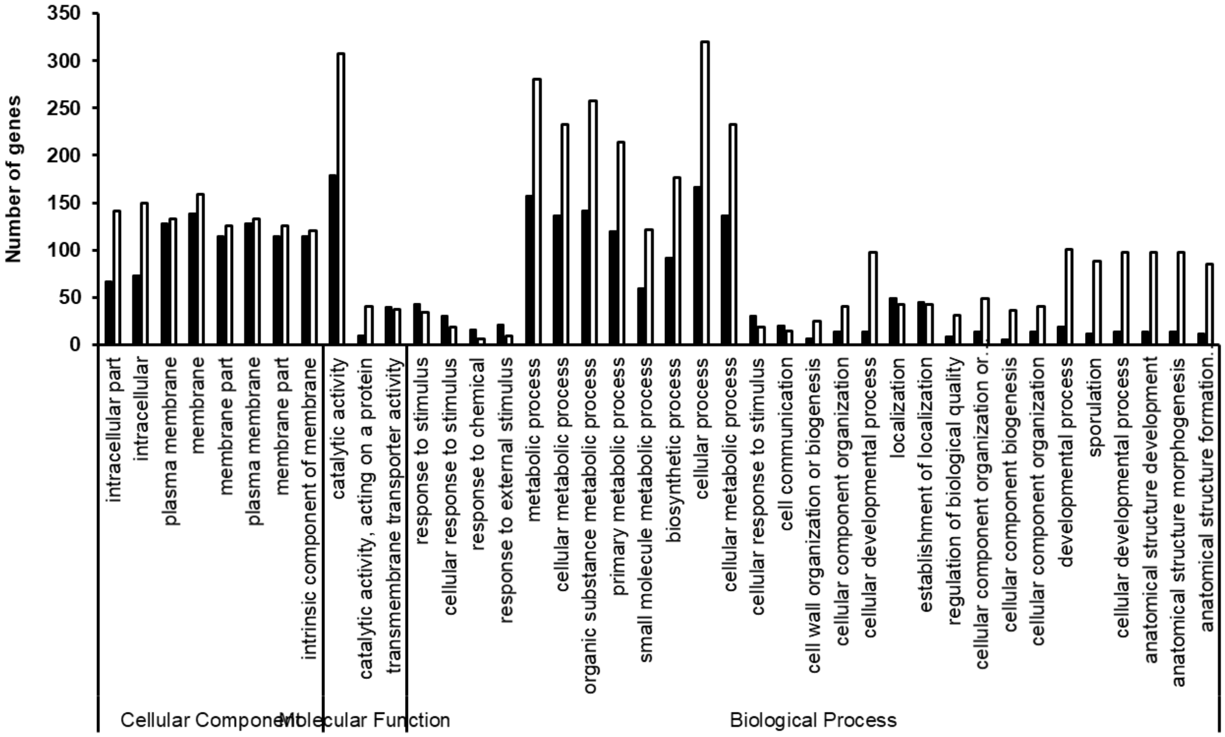

Suppl. Fig. 22. GO terms differentially expressed in Δmatrix during the interaction with PCL1606 (72 h). Black bars indicate induced GO terms while empty bars indicate repressed GO terms.

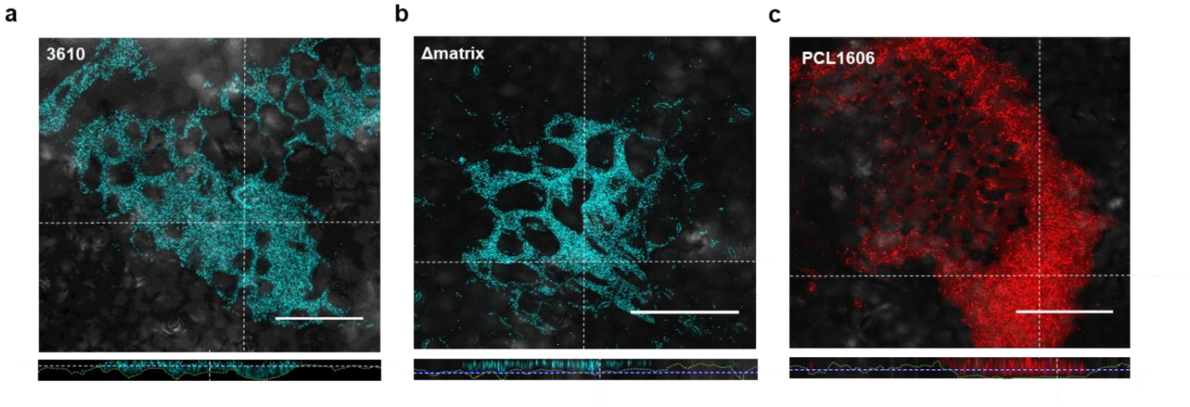

Suppl. Fig. 23. CLSM maximum projections and z axis slices at positions indicated by the discontinuous lines of the bacterial distribution of (a) 3610, (b) Δmatrix and (c) PCL1606 after 9 days of inoculation. *Bacillus* strains were labeled with CFP and PCL1606 was labeled with DsRed. Scale bar = 100 µm. Green dotted lines indicate the leaf surface.

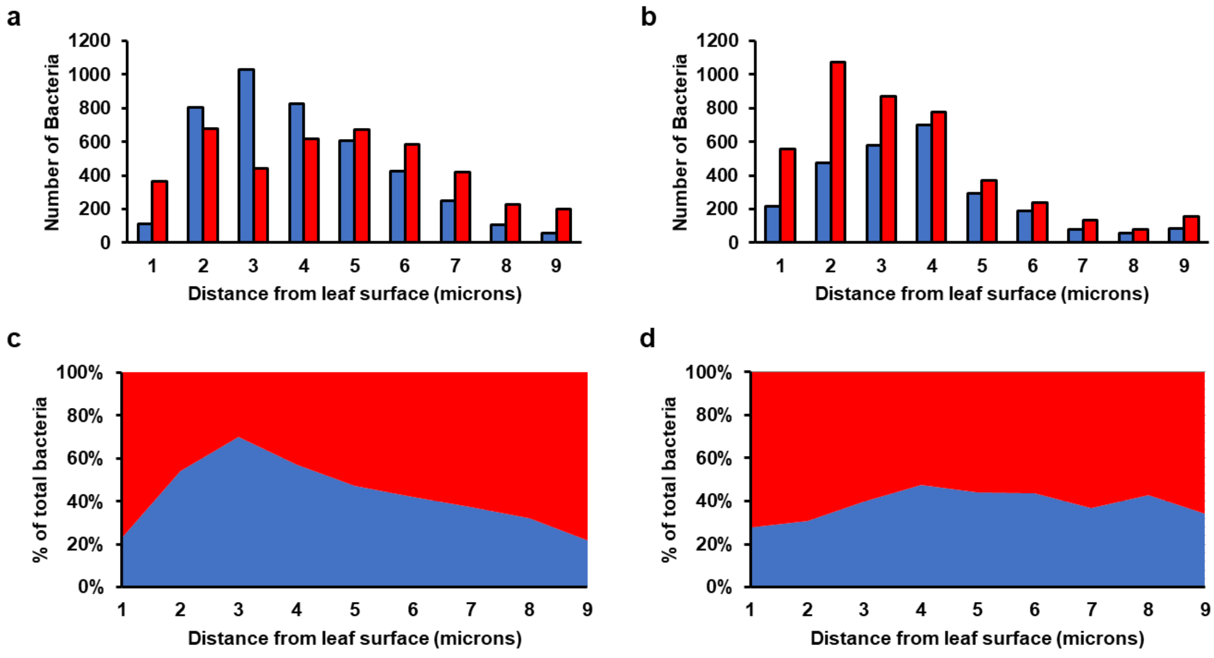

Suppl. Fig. 24. Bacterial distribution relative to melon leaf surfaces after 9 days post-inoculation. *Bacillus* species are shown in blue whereas PCL1606 is shown in red. (a and b) Number of bacteria at different distances (1 to 9 microns) from the leaf surface when (a) 3610 and PCL1606, and (b) Δmatrix and PCL1606 were co-inoculated. (c and d) Percentage of total bacteria at different distances from the leaf surfaces of interactions (c) 3610 – PCL1606 and (d) Δmatrix – PCL1606. Source data are provided as a Source Data file.

References

1. Branda SS, Gonzalez-Pastor JE, Ben-Yehuda S, Losick R, Kolter R. Fruiting body formation by Bacillus subtilis. *Proceedings of the National Academy of Sciences of the United States of America* **98**, 11621-11626 (2001).

2. Romero D, Aguilar C, Losick R, Kolter R. Amyloid fibers provide structural integrity to Bacillus subtilis biofilms. *Proc Natl Acad Sci U S A* **107**, 2230-2234 (2010).

3. Branda SS, Chu F, Kearns DB, Losick R, Kolter R. A major protein component of the Bacillus subtilis biofilm matrix. *Mol Microbiol* **59**, 1229-1238 (2006).

4. Grau RR*, et al.* A Duo of Potassium-Responsive Histidine Kinases Govern the Multicellular Destiny of Bacillus subtilis. *MBio* **6**, e00581 (2015).

5. Wang L, Grau R, Perego M, Hoch JA. A novel histidine kinase inhibitor regulating development in Bacillus subtilis. *Genes Dev* **11**, 2569-2579 (1997).

6. Cazorla FM*, et al.* Biocontrol of avocado dematophora root rot by antagonistic Pseudomonas fluorescens PCL1606 correlates with the production of 2-hexyl 5-propyl resorcinol. *Mol Plant Microbe Interact* **19**, 418-428 (2006).

7. Calderon CE, Perez-Garcia A, de Vicente A, Cazorla FM. The dar genes of Pseudomonas chlororaphis PCL1606 are crucial for biocontrol activity via production of the antifungal compound 2-hexyl, 5-propyl resorcinol. *Mol Plant Microbe Interact* **26**, 554-565 (2013).

8. Calderon CE, Ramos C, de Vicente A, Cazorla FM. Comparative Genomic Analysis of Pseudomonas chlororaphis PCL1606 Reveals New Insight into Antifungal Compounds Involved in Biocontrol. *Mol Plant Microbe Interact* **28**, 249-260 (2015).

9. Calderon CE, de Vicente A, Cazorla FM. Role of 2-hexyl, 5-propyl resorcinol production by Pseudomonas chlororaphis PCL1606 in the multitrophic interactions in the avocado rhizosphere during the biocontrol process. *FEMS Microbiol Ecol* **89**, 20-31 (2014).
