# Supplementary material for: The extracellular matrix protects Bacillus subtilis colonies from Pseudomonas invasion and modulates plant co-colonization
Source: Nat Commun. 2019 Apr 23;10:1919. doi: 10.1038/s41467-019-09944-x (PMC6478825; doi:10.1038/s41467-019-09944-x)
Supplement: Supplementary file 3 — Description of Additional Supplementary Files [file 41467_2019_9944_MOESM3_ESM.pdf]

Description of Additional Supplementary Files

File Name: Supplementary Data 1
Description: Differentially expressed genes in PCL1606 after interaction with Δmatrix. p-value 1 and < -1.

File Name: Supplementary Data 2
Description: Differentially expressed genes in Δmatrix after interaction with PCL1606. p-value 1 and < -1.

File Name: Supplementary Data 3
Description: Strains used in this work.

File Name: Supplementary Data 4
Description: Oligonucleotides used in this work.

File Name: Supplementary Movie 1
Description: Movie of the interaction between PCL1606 and 3610 by CLSM during 72 h.

File Name: Supplementary Movie 2
Description: Movie of the interaction between PCL1606 and Δmatrix by CLSM during 72 h.
