# Supplementary material for: The extracellular matrix protects Bacillus subtilis colonies from Pseudomonas invasion and modulates plant co-colonization
Source: Nat Commun. 2019 Apr 23;10:1919. doi: 10.1038/s41467-019-09944-x (PMC6478825; doi:10.1038/s41467-019-09944-x)
Supplement: Supplementary file 10 — Reporting Summary [file 41467_2019_9944_MOESM10_ESM.pdf]

nature research

Corresponding author(s): Romero, D

Last updated by author(s): Feb 25, 2019

Reporting Summary

Nature Research wishes to improve the reproducibility of the work that we publish. This form provides structure for consistency and transparency in reporting. For further information on Nature Research policies, see Authors & Referees and the Editorial Policy Checklist.

Statistics

For all statistical analyses, confirm that the following items are present in the figure legend, table legend, main text, or Methods section.

n/a	Confirmed	
☐	☒	The exact sample size (*n*) for each experimental group/condition, given as a discrete number and unit of measurement
☐	☒	A statement on whether measurements were taken from distinct samples or whether the same sample was measured repeatedly
☐	☒	The statistical test(s) used AND whether they are one- or two-sided<br>*Only common tests should be described solely by name; describe more complex techniques in the Methods section.*
☒	☐	A description of all covariates tested
☒	☐	A description of any assumptions or corrections, such as tests of normality and adjustment for multiple comparisons
☐	☒	A full description of the statistical parameters including central tendency (e.g. means) or other basic estimates (e.g. regression coefficient) AND variation (e.g. standard deviation) or associated estimates of uncertainty (e.g. confidence intervals)
☐	☒	For null hypothesis testing, the test statistic (e.g. *F*, *t*, *r*) with confidence intervals, effect sizes, degrees of freedom and *P* value noted<br>*Give P values as exact values whenever suitable.*
☒	☐	For Bayesian analysis, information on the choice of priors and Markov chain Monte Carlo settings
☒	☐	For hierarchical and complex designs, identification of the appropriate level for tests and full reporting of outcomes
☒	☐	Estimates of effect sizes (e.g. Cohen's *d*, Pearson's *r*), indicating how they were calculated

Our web collection on statistics for biologists contains articles on many of the points above.

Software and code

Policy information about availability of computer code

Data collection	RNA-seq data were collected using commercial softwares from Illumina. Confocal microscopy images were taken using commercial softwares from Leica. MALDI-IMS images were collected using a commercial software from Bruker Daltonics, while metabolomics data were obtained with the commercial software of Thermo Scientific for UPLC systems.
Data analysis	For RNA-seq data analysis, Raw reads were quality-trimmed with cutadapt. Then, trimmed reads were mapped against Pseudomonas chlororaphis PCL1606, Bacillus subtilis 3610 reference genomes using EDGE-pro software, which is specially designed to process prokaryotic RNA-Seq data. Quantification was also performed with EDGE-pro. Raw counts were normalized using the trimmed mean of M-values (TMM) method, implemented in NOISeq R package. Differential expression analyses were performed with DESeq2 package.<br><br>For MALDI-IMS data analysis Samples were analyzed using a Bruker Microflex MALDI-TOF mass spectrometer (Bruker Daltonics, Billerica, MA, USA) in positive reflectron mode, with 300 µm–400 µm laser intervals in X and Y directions, and a mass range of 100–3200 Da. Data obtained were analyzed using FlexImaging 3.0 software (Bruker Daltonics, Billerica, MA, USA).<br><br>For LC-MS/MS data analysis, raw spectra were converted to .mzXML files using MSconvert (ProteoWizard). MS1 and MS/MS feature extraction was performed with Mzmine2.3073. For MS1 spectra an intensity threshold of 1E5 and for MS/MS spectra of 1E3 was used. For MS1 chromatogram building a 10 ppm mass accuracy and a minimum peak intensity of 5E5 was set. Extracted Ion Chromatograms (XICs) were deconvoluted using the baseline cut-off algorithm at an intensity of 1E5. After chromatographic deconvolutiuon, XICs were matched to MS/MS spectra within 0.02 m/z and 0.2 min retention time windows. Isotope peaks were grouped and features from different samples were aligned with 10 ppm mass tolerance and 0.1 min retention time tolerance. MS1 features without MS2 features assigned were filtered out the resulting matrix as well as features which did not contain isotope peaks and which did not occur at least in 3 samples. After filtering gaps in the feature matrix were filled with relaxed retention time tolerance at 0.2 min but also 10 ppm mass tolerance. Finally, the feature table was exported as .csv file and corresponding MS/MS spectra as .mgf file. Contaminate features observed in Blank samples were filtered and only those whose relative abundance ratio blank to average in the samples was lower than 50% were considered for further analysis.

For molecular networking and spectrum library matching the .mgf file was uploaded to GNPS (gnps.ucsd.edu). For molecular networking, the minimum cosine score was set as 0.7. The Precursor Ion Mass Tolerance was set to 0.01 Da and Fragment Ion Mass Tolerance to 0.01 Da, Minimum Matched Fragment Peaks was set to 4, Minimum Cluster Size to 1 (MS Cluster off) and Library Search Minimum Matched Fragment Peaks to 4. When Analog Search was performed the Cosine Score Threshold was 0.7 and Maximum Analog Search Mass Difference was 100. Molecular networks were visualized with Cytoscape version 3.4.
For CLSM data analysis, image processing was performed using Leica LAS AF (LCS Lite, Leica Microsystems) and FIJI/ImageJ and Imaris version 7.6 (Bitplane) softwares.

For manuscripts utilizing custom algorithms or software that are central to the research but not yet described in published literature, software must be made available to editors/reviewers. We strongly encourage code deposition in a community repository (e.g. GitHub). See the Nature Research guidelines for submitting code & software for further information.

Data

Policy information about availability of data

All manuscripts must include a data availability statement. This statement should provide the following information, where applicable:

- Accession codes, unique identifiers, or web links for publicly available datasets
- A list of figures that have associated raw data
- A description of any restrictions on data availability

RNA-seq deposited at GEO database with accession code: GSE117802
Metabolomics data deposited at https://massive.ucsd.edu/ with the identifier MSV000082402

Field-specific reporting

Please select the one below that is the best fit for your research. If you are not sure, read the appropriate sections before making your selection.

☒ Life sciences ☐ Behavioural & social sciences ☐ Ecological, evolutionary & environmental sciences

For a reference copy of the document with all sections, see nature.com/documents/nr-reporting-summary-flat.pdf

Life sciences study design

All studies must disclose on these points even when the disclosure is negative.

Sample size	Sample-size calculation was not initially performed but a sufficient number of replicates were taken in order to get statistically confident results. In all the experiments performed standard deviation was calculated and Tukey's analysis was done when needed.
Data exclusions	No data were excluded from the study.
Replication	At least three replicates were taken in all the experiments performed. In all the cases the experiments were successfully replicated
Randomization	Randomization is not relevant in this case as our study is mainly focused in the interaction between two bacterial species
Blinding	Blinding is not relevant in this case as our study is mainly focused in the interaction between two bacterial species

Reporting for specific materials, systems and methods

We require information from authors about some types of materials, experimental systems and methods used in many studies. Here, indicate whether each material, system or method listed is relevant to your study. If you are not sure if a list item applies to your research, read the appropriate section before selecting a response.

Materials & experimental systems

n/a	Involved in the study	
☒	☐	Antibodies
☒	☐	Eukaryotic cell lines
☒	☐	Palaeontology
☒	☐	Animals and other organisms
☒	☐	Human research participants
☒	☐	Clinical data

Methods

n/a	Involved in the study	
☒	☐	ChIP-seq
☒	☐	Flow cytometry
☒	☐	MRI-based neuroimaging
